# Assessment of the Relationship between Clinical Manifestation and Pathogenic Potential of *Streptococcus pyogenes* Strains-Distribution of Genes and Genotypes of Toxins

**DOI:** 10.3390/biomedicines10040799

**Published:** 2022-03-29

**Authors:** Tomasz Bogiel, Alicja Domian, Zuzanna Dobrzyńska, Agnieszka Mikucka, Eugenia Gospodarek-Komkowska

**Affiliations:** 1 Microbiology Department, Ludwik Rydygier Collegium Medicum in Bydgoszcz, Nicolaus Copernicus University in Torun, 9 M. Sklodowska-Curie Street, 85-094 Bydgoszcz, Poland; alicjadomian@abs.umk.pl (A.D.); zuza.dobrzynska@abs.umk.pl (Z.D.); a.mikucka@cm.umk.pl (A.M.); gospodareke@cm.umk.pl (E.G.-K.); 2 ALAB Laboratories Ltd., Based in Warsaw, 22/30 Stepinska Street, 00-739 Warsaw, Poland

**Keywords:** exotoxins, pyrogenic exotoxins, *speA*, *speB*, *speC*, *speH*, *speJ*, *speK*, *Streptococcus pyogenes*, toxins, virulence, virulence factors, virulence factors genes

## Abstract

*Streptococcus pyogenes* is one of the most important species among beta-haemolytic streptococci, causing human infections of different localization. It is isolated from clinical specimens relatively frequently. In this study, the frequency and co-occurrence of toxin genes (*speA*, *speB*, *speC*, *speH*, *speJ*, *speK*) among 147 *S. pyogenes* strains were evaluated, using real-time PCR. In addition, the relationship between the occurrence of these genes and the origin of *S. pyogenes* strains from selected clinical material was assessed. The *speB* gene was present with the highest incidence (98.6%), while the *speK* gene was the least frequent (8.2%) among the tested strains. Based on the presence of the detected genes, the distribution of 17 genotypes was determined. The most common (21.8%), was *speA* (−) *speB* (+) *speC* (−) *speH* (−) *speJ* (−) *speK* (−) genotype. Furthermore, significant variation in the presence of some genes and genotypes of toxins in *S. pyogenes* strains isolated from different types of clinical material was found. There is a considerable variety and disproportion between the frequency of individual genes and genotypes of toxins in *S. pyogenes* strains. The relationship between the origin of *S. pyogenes* isolates and the presence of toxins genes indicates their pathogenic potential in the development of infections of selected localization.

## 1. Introduction

*Streptococcus pyogenes* is classified as one of the most dangerous pathogens that causes infections only in humans. These Gram-positive bacteria mainly colonize the mucous membrane of the throat and skin, contributing to various forms of infections and their complications. These infections, which can be non-invasive or invasive, especially involve the respiratory tract, skin and soft tissue, and bloodstream infection [1]. Despite a progress in medicine, *S. pyogenes* can still be life-threatening. Therefore, knowledge of pathogenic potential of the species and supervision of diseases caused by these microorganisms is of high importance. This constitutes the basis for their control and prevention [2].

*S. pyogenes* strains determine their pathogenicity by numerous virulence factors synthesis. These include surface proteins (e.g., M protein, lipoteichoic acids, fibronectin-binding protein), enzymes (e.g., hyaluronidases, streptokinases), hemolysins (streptolysins S and O), or produced extracellular substances (e.g., cytolytic toxins, pyrogenic exotoxins) [2,3]. They play an important role in colonization, provide a line of defense against the host immune system, and influence the course of disease [2]. In this research we focused on pathogenic potential resulting from the following toxins synthesis: A, B, C, J, H, and K. These substances, encoded by *speA*, *speB*, *speC*, *speJ*, *speH*, and *speK* genes, belong to superantigens. They have the ability to stimulate the activity of T and B lymphocytes of the colonized organism [4,5]. The immune response is so strong that there is an excessive release of inflammatory mediators including INF-γ (interferon gamma) and TNF-α (tumor necrosis factor alpha). As a consequence, effusions, extensive organ damage, and the onset of shock can be observed [2,6]. In addition, superantigens can have mitogenic and pyrogenic effects, and also inhibit immunoglobulin synthesis. This distorts the proper functions of the host immune system, which promotes the entry of purulent streptococci into human cells [4,6]. Among the toxins mentioned above, SpeB serves as a potent cysteine protease involved profoundly in the virulence of *S. pyogenes* [2,3,5].

Improving our knowledge of the involvement of streptococcal toxins will allow us to determine the pathogenic potential of group A *Streptococcus* (GAS) strains, make a diagnosis easier and faster, or even prognose the course of infection. This may enable the implementation of appropriate targeted therapy earlier. This, in turn, translates into the length and efficiency of healing process by shortening hospitalization time with minimizing side effects of treatment. In the long term, this may also contribute to reducing antibiotic resistance of *S. pyogenes* strains. Hence, the aim of this study was to assess, with the application of real-time PCR, the frequency of streptococcal toxins genes: *speA*, *speB*, *speC*, *speJ*, *speH*, and *speK* and to determine the genotypes distribution of *S. pyogenes* strains. An additional purpose of this study was to determine the virulence potential of individual *S. pyogenes* strains derived from different body sites to evaluate a relation between these genes and/or genotypes presence and the origin of *S. pyogenes* strains from specific types of clinical material.

## 2. Results

The conducted research enabled us to show the wide variety in the percentages of the examined genes and the distribution of genotypes, as shown in Table 1. The *speB* gene was observed with the highest frequency-145 (98.6%) strains, while the *speK* was the least frequent gene-12 (8.2%) strains.

The examples of amplification curves for each particular gene are presented in Supplementary Material–Figure S1, while the high resolution melting curves obtained to confirm amplification specificity can be found in Figure S2.

Based on the detected genes, 17 genotypes (A–Q) among the studied *S. pyogenes* strains were determined. The A genotype, which was devoid of each of the detected genes except the *speB* gene, was the most common-32 (21.8%) strains. Four (2.7%) strains presented distinctive genotypes (N–Q). The distribution of genes and genotypes among the *S. pyogenes* strains was statistically compared with their theoretical distribution. In each case, the *p*-value was below 0.001, except the distribution of the *speC* gene (*p* = 0.458).

A statistically significant relationship was noted between the genes: *speA* and *speC* (*p* = 0.002), *speA* and *speH* (*p* = 0.013), *speA* and *speJ* (*p* < 0.001), *speC* and *speH* (*p* < 0.001), *speC* and *speJ* (*p* = 0.037), and *speH* and *speJ* (*p* < 0.001).

The *speA* and *speJ* genes were most common among the strains isolated from throat swabs–amongst 6 (31.6%) strains and 14 (33.3%) strains, respectively. In contrast, *speB* (60 strains, 41.4%), *speC* (31 strains, 44.9%), *speH* (20 strains, 52.6%) and *speK* (5 strains, 41.7%) genes dominated amongst the strains isolated from wound swabs. The percentages of GAS strains with the presence of particular genes, which were isolated from selected types of clinical material, are presented in Table 2.

There was a statistically significant relation between the occurrence of: the *speA* gene and the origin of *S. pyogenes* strains from wound swabs (*p* = 0.033); the *speJ* gene and *S. pyogenes* strains origin from throat swabs (*p* = 0.032); and the *speH* gene and the origin of *S. pyogenes* strains from purulent material (*p* = 0.012).

The A–D genotypes were the most prevalent among the strains isolated from wound swabs (43.8%, 52.0%, 47.8%, and 50.0%, respectively). In turn, genotypes E and F (both 38.5%) were dominant in strains isolated from throat swabs. The percentages of GAS strains with specified genotype (A–F), which were isolated from selected types of clinical material, are shown in Table 3.

The relationship between: the presence of A genotype, and the origin of GAS strains from ulcer swabs (*p* = 0.002), the presence of B genotype, and the origin of GAS strains from purulent material (*p* = 0.001), and the presence of E genotype, and the origin of GAS strains from wound swabs (*p* = 0.024), has been demonstrated.

## 3. Discussion

*S. pyogenes* belongs to the group of bacterial pathogens contributing to the development of human infections, which significantly differ in location, course, prognosis, and mortality [7]. Therefore, it is important to conduct research on the pathogenic potential of *S. pyogenes*, consisting in the detection of the most important virulence factors or genes encoding them and the assessment of their occurrence depending on the location and form of infection.

The mentioned virulence factors genes of *S. pyogenes* are mostly pyrogenic toxins and proteases, among others: *speA*, *speB*, *speC*, *speJ*, *speH*, and *speK*, which encode streptococcal toxins designated as speA, speB, speC, speJ, speH, and speK, respectively. Streptococcal pyrogenic exotoxins are virulence factors produced exclusively by *S. pyogenes*. They are encoded by genes located on bacterial chromosomes or prophages [8,9]. Not all the genes of toxins are present in each GAS strain. Therefore, differences in the frequency of particular genes encoding these virulence factors can be observed and implicate the course of the disease and prognostic factors [10].

In the present research, the frequency of the *speA* gene was noted in 19 (12.9%) of the examined *S. pyogenes* isolates. There was also a statistically significant difference in the distribution of this gene between the studied GAS strains. In Yu and Ferretti [11] research, including 302 *S. pyogenes* isolates from patients from different countries, the percentage of strains with the presence of the *speA* gene was 14.9%. This result was similar to that obtained in our own research. The studies presented by Szczypa et al. [12] indicated the frequency of the *speA* gene in the examined strains at the level of 24.4%. The number of *S. pyogenes* isolates used in the above-mentioned research was, however, much lower (41 strains) than in the presented results of our own research. In the study by Li et al. [13], the gene determining the presence of pyrogenic toxin A was present in 34.3% of the tested isolates. This study included 271 GAS strains. The analysis of the results of our own and other authors, concerning the frequency of the *speA* gene in *S. pyogenes* strains, showed slight differences between the presence and the absence of this gene in terms of different forms of infections caused by the studied isolates.

The presence of the *speB* gene in the studied *S. pyogenes* strains was confirmed among 145 (98.6%) isolates in our own research. There was also a statistically significant difference in the distribution of the encoding speB gene between the studied GAS strains (*p* < 0.001). Tyler et al. [14] noted the presence of the *speB* gene in 99.3% of *S. pyogenes* strains, causing invasive and non-invasive diseases. The result is relatively close to the results of our study. Interestingly, research presented by Wu et al. [15] showed the presence of the gene encoding speB only in 81.0% of GAS strains isolated from patients with scarlet fever and amongst 72.4% of strains isolated from patients with *acute pharyngitis* and *tonsillitis*. In the available literature, it is also possible to observe a higher percentage of GAS isolates carrying the *speB* gene in patients diagnosed with invasive forms of *S. pyogenes* infections [16]. Despite slight differences, the frequency of the *speB* gene was always high in the available literature.

In the presented studies, the frequency of the *speC* gene was found in almost half of the examined *S. pyogenes* strains (46.9%), and there was no statistically significant difference between the number of GAS strains with and without the gene encoding speC. In the research work of Yu and Ferretti [17], which included 315 isolates, similarly to our own studies, the gene encoding pyrogenic toxin C was present in 50.8% of *S. pyogenes* strains. In the study by Maripuu et al. [18], the presence of the *speC* gene was significantly higher than that recorded on the basis of our own research results. This gene was detected among 66.3% isolates causing bacteremia, Streptococcal Toxic Shock Syndrome (STSS), and *erysipelas* (the cited analysis included 92 GAS strains). This might indicate a greater share of strains with such a pathogenic potential in invasive infections [2]. The occurrence of *speC* was differently assessed by Li et al. [13]. The presence of that gene in the strains tested by them reached 91.1%. The fact that as many as 219 isolates, out of 271 included in the study, were derived from patients with scarlet fever, could have an impact on such a high percentage of strains with *speC* gene.

In our research, the percentage of *S. pyogenes* strains with the *speJ* gene was 28.6%. Statistically significant differences were also observed between the number of GAS strains with and without the gene encoding *speJ*. A similar result (32.7%) was obtained by Friães et al. [19], although the number of strains used by them for the analysis was almost three times higher. Among the genes of pyrogenic exotoxins investigated by them and encoded on the bacterial chromosome, i.e., *speJ*, *speG*, and *smeZ*, the *speJ* gene was the least frequent. In the study by Li et al. [20], the *speJ* gene was present among isolates with a frequency of 22.2%. The researchers detected 11 genes encoding superantigens, of which *speJ* belonged to the group of the three rarest genes encoding selected superantigens. On the other hand, Berman et al. [21] found the *speJ* gene only in 11 (14.3%) strains, but the number of the tested isolates was over half lower than that in our own research. Taking into account 11 genes encoding superantigens they detected, *speJ* was relatively rare, while six other genes, i.e., *speG*, *smeZ*, *speI*, *speC*, *speA*, and *speH*, were found more often.

Compared to the *speJ* gene, the frequency of *speH* and *speK* genes in our own research differed to a greater extent from the results of other authors’ studies. In our studies, the *speH* gene was found in 38 (25.9%) examined strains, and the *speK* gene in 12 (8.2%). Statistically significant differences were observed between the number of GAS strains with and without the gene encoding *speH* and *speK*. The *p*-value was below 0.001 for both genes distribution. Friães et al. [19] revealed the presence of the *speH* and *speK* genes in 82 (17.1%) and 118 (24.6%) GAS strains, respectively. Taking into account all the superantigen genes that they studied, which are encoded within prophages, *speH* and *speK* occurred relatively rarely. In Li et al. studies [20], the *speH* gene was noted in 155 (52.2%) strains of *S. pyogenes*, and the *speK* gene in only 2.0% of the isolates. It should be noted that *speK* was the least frequently detected gene, which was consistent with the results obtained in our own study. In the study by Berman et al. [21], the percentage of GAS strains with the *speH* gene was 36.4%. The researchers did not detect the *speK* gene in any of the *S. pyogenes* strains and the results obtained by them were similar to those developed by Li et al. [20].

Comparing the results of this study with the results of the above authors, it can be concluded that the reason for the differentiation of percentage of individual genes of toxin may be the very specificity of GAS strains from patients from different geographic regions, age of patients, or, particularly, symptoms of infection occurring in patients, but the above-mentioned aspects were not the subject of research of this work.

Of note, the *speA*, *speC*, *speH*, and *speK* genes are located within the prophages, which also had a key influence on the differences in their frequency [21,22]. According to the available literature, the frequency of pyrogenic toxins genes is also influenced by the presence of particular *emm* types. For example, the genes *speA* and *speJ* are found much more often in strains of the *emm1* type. The *speC* gene is most common in GAS strains of the *emm28* type, the *speH* gene in the GAS strains of the *emm12* type, while the *speK* gene is associated with, among others, *emm2* and *emm3* types [18,23]. However, the emm types distribution of *S. pyogenes* was not the subject of this study.

Having data on the frequency of genes encoding virulence factors, it is possible to determine the patho-genetic profile (genotypes) of the studied GAS strains. In our own research, 17 different genotypes were observed. Their distribution differed significantly from the theoretical one, as indicated by the *p*-value, which was below 0.001. Namely, the number of *S. pyogenes* strain groups to which specific genotypes were assigned was quite diverse.

Compared to researches of other authors, the number of grouped genotypes was relatively small. This was due to the much greater number of virulence factor genes that have been detected in other studies. The more genes tested, the greater the diversity of the gene profiles. An important reason for obtaining a different number of genotypes was also the matter of the diversity of *S. pyogenes* strains used for the study. In our own research, these strains were isolated from many types of clinical material, but throat and wound swabs were the most common. This means that the isolated GAS strains could show some similarity in the presence of the *speA*, *speB*, *speC*, *speJ*, *speH*, and *speK* genes and, as a consequence, the convergence of genotypes. Moreover, most of the strains were isolated within one hospital, which probably resulted in a limited degree of possible geographic differentiation of the isolates.

Helal et al. [24] revealed large diversity of the genotypes they identified. They recorded 33 genotypes among only 38 *S. pyogenes* strains. They put together these genotypes on the basis of the genes *speA*, *speC*, *speG*, *speH*, *speI*, *speJ*, *speK*, and *ssa*. Despite the small number of GAS strains studied by them, the number of genotypes was almost twice as high as in our own research. Abraham and Sistla [25] detected as many as 71 gene profiles, defined on the basis of the genes *speA*, *speC*, *speG*, *speH*, *speI*, *speJ*, *speK*, *speL*, *speM*, *smeZ*, and *ssa*, among 206 *S. pyogenes* strains. They isolated these strains from clinical material derived from patients with both non-invasive and invasive infections. It is noteworthy that the authors found the presence of 66 *emm* types of *S. pyogenes*, and showed a relationship between several of them and specific genotypes. The number of genotypes obtained during the research is undoubtedly influenced by the *emm* types among the *S. pyogenes* strains. Different *emm* types may have the same genotype and, vice versa, one *emm* type may be associated with different genotypes. Another factor contributing to the existence of different genotypes was horizontal gene transfer. The acquisition or loss of prophage by a bacterial cell favors its genetic variability. This translates into the differences in the frequency of particular gene profiles in the GAS population, and into intraspecific variation by generating new genotype variants [8,21,25,26].

Our own research shows that in *S. pyogenes* strains, the genotype *speA* (−) *speB* (+) *speC* (−) *speJ* (−) *speH* (−) *speK* (−) (genotype A) was the most common (21.8%). This does not mean, however, that they only produced speB toxin in the infections pathomechanism. They could have other virulence factors genes that were not included in this study. When analyzing the presence of the *speA*, *speB*, *speC*, *speJ*, *speH*, and *speK* genes, the percentage of genotype A in the research of other authors was negligible. Meisal et al. [27], who established gene profiles of 262 isolates causing invasive infections, detected genotype A in 15 (5.7%) GAS strains. Ibrahim et al. [28] and Maripuu et al. [18] found this genotype in only one strain (11.1% and 1.1%, respectively). This shows that the *speB* gene co-exists more often with the genes of pyrogenic exotoxins. Our research shows that it was most often the *speC* gene, which was confirmed by a fairly high percentage of genotype B (25 strains, 17.0%), C (23 strains, 15.6%), and F (13 strains, 8.8%). The simultaneous presence of these two genes was found in a total of 69 (46.9%) strains of *S. pyogenes*. However, there was no statistically significant relationship between the *speC* and *speB* genes (*p* = 0.531). A similar result was obtained by Commons et al. [23]. Almost half of the strains examined by them (53 isolates, 49.5%) were positive for the *speB* and *speC* genes, but the co-existence of the *speB* and *speG* genes was the most common (96 strains, 89.7%).

The genes investigated in the present study more often co-exist with genes of other virulence factors that have not been analyzed in our research. The *speJ* gene is usually associated with the *smeZ* gene. Mostly, the *speH* and *speI* genes are also detected because they are encoded within the genetic material of the same bacteriophage φ370.2. On the other hand, the *speK* gene is usually associated with the *speA* gene [19,28]. In the case of our own research, only in two strains of *S. pyogenes* the simultaneous presence of the *speA* and *speK* genes was found. Furthermore, no statistically significant correlation between them was observed (*p* = 0.963), in contrast to the *speA-speC*, *speA-speH*, and *speA-speJ* gene pairs for which the *p*-value reached 0.002, 0.013, and <0.001, respectively.

In our own research, the percentage of N-Q genotypes was the lowest. They were found once among the tested GAS strains. In the study by Berman et al. [21], the rarest gene profiles were completely different. The fact that the *speA* (−) *speB* (+) *speC* (−) *speJ* (−) *speH* (+) *speK* (−) genotype (genotype G in our study), found by them in one strain, was detected by us in 9 (6.1%) GAS strains, is the evidence of the varied distribution of gene profiles among *S. pyogenes* strains.

*S. pyogenes* strains are isolated from many types of clinical material, such as throat and wound swabs or purulent material and blood [7,29]. By detecting genes encoding virulence factors it can be assessed whether their presence is related to the origin of GAS strains from a specific clinical material. In our study, the *speA* and *speJ* genes were most common (31.6% and 33.3%, respectively) among the strains isolated from throat swabs. Moreover, a statistically significant correlation was found between the presence of the *speJ* gene and the origin of GAS strains from these clinical materials (*p* = 0.032). The throat swab is usually taken in the case of *pharyngitis* or *tonsillitis*, which are classified as non-invasive infections [3]. Altun and Mericli Yapıcı [30], who conducted studies among 200 patients with *pharyngitis* and *tonsillitis*, isolated only 15 GAS strains. The *speA* gene was present in three, and the *speJ* gene was present in only two of them. However, their research did not assess the relationship between the presence of the detected genes and the origin of *S. pyogenes* strains. Hamzah et al. [31] found the *speA* and *speJ* genes more frequently in GAS strains isolated from patients with non-invasive rather than invasive infections (65.0% vs. 35.0% for *speA* and 63.6% vs. 36.4% for *speJ*). According to them, this could be due to the overall greater number of GAS isolates that came from people with non-invasive infections. Detection of *speA* or *speJ* genes in GAS strains may also be associated with the potential development of invasive infections. Kittang et al. [32] demonstrated a relationship between these genes and the invasiveness of infections. In patients, from whom they isolated *S. pyogenes* strains, the following infections occurred: necrotizing fasciitis, STSS, *pneumonia*, *meningitis*, and *peritonitis*.

Although the *speB*, *speC*, *speH*, and *speK* genes, as well as A-D genotypes, dominated in strains isolated from wound swabs, no statistically significant correlation was found between the occurrence of these genes and gene profiles, and the origin of GAS strains from wound swabs. This could be due to the fact that the examined *S. pyogenes* strains, in general, were most often detected in this type of clinical material. The study by Strus et al. [9] shows that there is a relationship between the presence of the *speH* gene and the origin of the strains from this type of clinical material and wound infections in the patient. They explain that the reason for differences between the occurrence of superantigen genes and the type of clinical material and the form of infection, was the isolation of *S. pyogenes* strains from people from different countries and regions. On the other hand, in our own research, we found a correlation between the presence of the *speH* gene and the origin of the strains from purulent material (*p* = 0.012), and also the relation between the B genotype (in which the *speH* gene is found) and the origin of the strains from this type of material (*p* = 0.001). Purulent material culture is usually associated with an infection of the patient’s skin and subcutaneous tissue [33]. Therefore, it can be assumed that GAS strains with the *speH* gene contributed often to such infections.

There was also a relationship between A genotype and the origin of the strains from ulcer swabs. This may indicate that GAS strains with the *speB* gene only are a probable etiological factor of infections in patients with diabetes (diabetic foot infection) or patients with venous ulcers [34,35].

In conclusion, the results of this study show the diversity of *S. pyogenes* strains with regard to the presence of the *speA*, *speB*, *speC*, *speJ*, *speH*, and *speK* genes and their genotypes. Comparing our results with the available literature, the different values of the percentages of detected genes, as well as the number and percentages of gene profiles of the studied strains, were obtained, which confirms the validity of the above statement. The demonstrated relationships between the presence of some of the detected genes and genotypes, and the origin of *S. pyogenes* strains from specific types of clinical material, indicate that GAS strains isolated from them may be responsible for the occurrence of infection with a specific clinical manifestation. Different types of clinical material for microbiological investigation were used in the present study allowing GAS strains growth. It allowed for the assessment of their genetic diversity in relation to the origin of the isolates. Moreover, the assessment of the frequency of occurrence of the detected genes and the determination of the *S. pyogenes* strains genotypes, allowed for the evaluation of their relative pathogenic potential. The detection of exotoxins genes, as well as other virulence factors, may also be useful in the development of vaccines. Based on these genes, the virulence factors of the specific GAS strains can be determined. The most common of them may be a potential antigen necessary to induce the immune response in humans, which requires further and more detailed studies [36].

## 4. Materials and Methods

### 4.1. Origin of the Strains and Their Selection Criteria

Initially, the study involved 168 clinical isolates of *S. pyogenes*, which were isolated between 2008 and 2020, mainly from wound swabs (38.7%), throat swabs (19.6%), and purulent material (11.9%) collected for routine diagnostic purposes. These strains came from the collection of the Microbiology Department of Ludwik Rydygier Collegium Medicum in Bydgoszcz Nicolaus Copernicus University in Toruń, Poland. The tested GAS strains were generally isolated from patients from surgical departments (32.1%) and outpatient clinics (15.5%). The detailed origin of the isolates included into the study is presented in the Supplementary Material: Tables S1 and S2.

Due to the fact that some GAS strains were derived from the same patients, 147 strains were eventually used to perform gene and genotype distribution calculations. Each of them was isolated from a different patient. An example of a gel showing PFGE patterns for the selected group of strains used to exclude duplicate isolates (data not shown) is shown in the Supplementary Material (Figure S3).

The reference *S. pyogenes* strains, derived from the German Collection of Microorganisms and Cell Cultures (DSMZ), served as positive control for a particular gene detection.

### 4.2. Bacterial Culture and Strain Identification

The tested and reference strains were plated on Columbia Agar with 5% sheep blood (Becton Dickinson, Franklin Lakes, NJ, USA). The culture was carried out at 37 °C for 18–20 h. The species affiliation of the tested and reference strains was confirmed by the (Matrix-Assisted Laser Desorption/Ionization Time-of-Flight Mass Spectrometry) MALDI-TOF MS method, using the MALDI Biotyper system (Bruker Daltonics GmbH & Co. KG, Bremen, Germany). This procedure was conducted according to the manufacturer’s protocol.

### 4.3. Bacterial Genomic DNA Isolation

Genomic DNA was isolated from the *S. pyogenes* strains in accordance with the instruction at the Centers for Disease Control and Prevention (CDC) website. In order to confirm the DNA isolation accuracy, the concentration of all DNA samples was evaluated spectrophotometrically (Photometer, Eppendorf, Germany). The DNA samples were stored at −20 °C before their further use in real-time PCR for the presence of the selected virulence factors genes evaluation.

### 4.4. Virulence Factor Genes Detection

The presence of the following genes: *speA*, *speB*, *speC*, *speH*, *speJ*, and *speK* was determined by real-time PCR method in the LightCycler 480 II Instrument (Roche Diagnostics, Basel, Switzerland). Positive and negative controls were used simultaneously and each time. The DNA isolated from *S. pyogenes*: DSMZ 20565 strain (for the *speA* and *speJ* genes), DSMZ 25932 strain (for the *speB*, *speC*, and *speH genes*) and DSMZ 11728 strain (for *speK* gene) served as PCR positive control. Reactions were performed with the application of molecular biology grade sterile water (EURx, Gdansk, Poland), F and R primers [9] (Genomed, DNA Sequencing and Oligonucleotide Synthesis Laboratory, Institute of Biochemistry and Biophysics of the Polish Academy of Sciences, Warsaw, Poland described in Table 4), and the 5x HOT FIREPol^®^ EvaGreen^®^ HRM Mix (no ROX) reaction mixture (Solis BioDyne, Estonia). The instruction attached to the reaction mixture was followed: the reaction volume of one sample was 20 µL with 4 µL of HRM Mix added to reach the final concentration of 1x, both primers were used at the final concentration of 200 nM (5 µL of each at the initial concentration of 1 µM), the remaining volume was water-5 µL and DNA template (1 µL). The amplification program was initially optimized for a particular gene and finally consisted of: initial activation at 95 °C for 5 min, followed by 45 cycles (50 for *speB* gene) of amplification, each consisting of 10 s at 95 °C, 20 s (10 s for *speJ* and *speK* genes) at the annealing temperature (see Table 4), followed by 72 °C for 20 s (60 s for *speH*, *speJ*, and *speK* genes) (for the amplification results see Figure S1).

After the amplification reaction, high resolution melting curves protocol was applied (95 °C for 5 s, 65 °C for 60 s, and constant heating until reaching 97 °C, ramp rate 0.11 °C/s with 5 read-outs per each °C) and the real-time PCR products specificity was checked (for the melting curves results see Figure S2).

To verify sizes of the amplification products for the particular genes and prove the specificity of the applied real-time PCR methodology, conventional PCR and electrophoresis were applied. Their results, as the examples of the gel pictures showing electrophoretic resolution of targeted and specific amplification of bacterial DNA, were included into Supplementary Material (Figures S4 and S5). It visualizes, separately, the amplicons of the particular genes for the selected reference strains: amplification of *speA* gene (200 bp), *speH* gene (630 bp), *speJ* gene (639 bp) (Figure S4), and *speB* gene (300 bp), *speC* gene (246 bp), and *speK* gene (564 bp) (Figure S5).

### 4.5. Statistical Analysis

In order to compare the distribution of genes and genotypes among the tested *S. pyogenes* strains, with the theoretical distribution, the Chi-Square Goodness of Fit Test with α ≤ 0.05 was carried out. The GNU PSPP (data analysis software system) program was used for this purpose. Whereas, in order to assess the relationship between the presence of genes and genotypes, and the origin of *S. pyogenes* strains from specific types of clinical material, the Chi-Square Test of Independence with α ≤ 0.05 was applied. The calculations were performed in the STATISTICA 13.3 (data analysis software system) program.

## 5. Conclusions

Among the clinical *S. pyogenes* strains, there is considerable diversity in the occurrence of toxin genes. The fact that the *speB* gene and the *speA* (−) *speB* (+) *speC* (−) *speJ* (−) *speH* (−) *speK* (−) genotype dominated in the studied GAS strains, might be related with the preferential coding of this toxins on the bacterial chromosome of this species strains. The predominance of the *speA* and *speJ* genes in strains isolated from throat swabs could potentially indicate a possible change in the clinical manifestation of the infection, from initially local to systemic. The relationship between the origin of *S. pyogenes* strains from purulent material and the more frequent occurrence of the *speH* gene among these strains indicates their molecular potential to cause skin and soft tissue infections.

Taking into account the pathogenic potential of a particular *S. pyogenes* strain and knowing the role of a particular toxin in the pathogenesis of *S. pyogenes* infections, we might be allowed to foresee the course of the infection. With the application of real-time PCR for particular toxins genes detection, as a standard microbiological procedure in the future, we may also be able to diagnose the infection easier, administer targeted antimicrobial therapy faster, and eventually decrease patients’ mortality rate.

## Figures and Tables

**Table 1:** The percentages of genes presence and genotype distribution among the examined *S. pyogenes* strains (*n* = 147); (+)–gene presence confirmed, (−)–lack of a particular gene.

Gene/Assigned Genotype Name	A	B	C	D	E	F	G	H	I	J	K	L	M	N	O	P	Q	*n*	%
*speB*	+	+	+	+	+	+	+	+	+	+	+	+	−	+	+	+	+	145	98.6
*speC*	−	+	+	−	−	+	−	+	+	−	−	−	−	+	−	−	+	69	46.9
*speJ*	−	−	−	+	+	+	−	−	−	−	−	−	−	+	+	−	−	42	28.6
*speH*	−	+	−	−	−	−	+	+	−	−	−	−	−	−	−	+	−	38	25.9
*speA*	−	−	−	−	+	−	−	−	−	+	−	+	−	+	−	−	+	19	12.9
*speK*	−	−	−	−	−	−	−	+	+	+	+	−	−	−	+	+	−	12	8.2
*n* = 147	32	25	23	14	13	13	9	3	3	2	2	2	2	1	1	1	1		
%	21.8	17.0	15.6	9.5	8.8	8.8	6.1	2.0	2.0	1.4	1.4	1.4	1.4	0.7	0.7	0.7	0.7		

**Table 2:** Occurrence of the virulence factor genes with respect to the origin of *S. pyogenes* strains from selected types of clinical material.

Clinical Specimen Type	Gene											
	*speA*		*speB*		*speC*		*speH*		*speJ*		*speK*	
	*n* = 19	%	*n* = 145	%	*n* = 69	%	*n* = 38	%	*n* = 42	%	*n* = 12	%
Wound swab	3	15.8	60	41.4	31	44.9	20	52.6	12	28.6	5	41.7
Throat swab	6	31.6	31	21.4	14	20.3	8	21.1	14	33.3	3	25.0
Purulent material	2	10.5	18	12.4	12	17.4	9	23.7	2	4.8	0	0.0
Ulcer swab	2	10.5	10	6.9	0	0.0	0	0.0	3	7.1	0	0.0
Blood samples	1	5.3	9	6.2	3	4.3	1	2.6	2	4.8	2	16.7
Nose swab	1	5.3	7	4.8	5	7.2	0	0.0	3	7.1	1	8.3

**Table 3:** The distribution of genotypes A–F with respect to origin of *S. pyogenes* strains from selected types of clinical material.

Clinical Specimen Type	Genotype											
	A		B		C		D		E		F	
	*n* = 32	%	*n* = 25	%	*n* = 23	%	*n* = 14	%	*n* = 13	%	*n* = 13	%
Wound swab	14	43.8	13	52.0	11	47.8	7	50.0	1	7.7	3	23.1
Throat swab	3	9.4	3	12.0	4	17.4	4	28.6	5	38.5	5	38.5
Purulent material	2	6.3	8	32.0	4	17.4	1	7.1	1	7.7	0	0.0
Ulcer swab	7	21.9	0	0.0	0	0.0	1	7.1	2	15.4	0	0.0
Blood samples	4	12.5	1	4.0	0	0.0	0	0.0	0	0.0	1	7.7
Nose swab	1	3.1	0	0.0	2	8.7	0	0.0	1	7.7	2	15.4

**Table 4:** The real-time PCR primers [9] specification and the annealing temperature applied in PCR amplification.

PCR Primer Name	Gene Detected	Primer Sequence 5′ → 3′	Tm (°C)	Annealing Temperature (°C)	Product Size (bp)
*speA*-F	*speA*	CTTAAGAACCAAGAGATGGC	49.7	52	200
*speA*-R		ATAGGCTTTGGATACCATC	46.8		
*speB*-F	*speB*	TTCTAGGATACTCTACCAGC	49.7	55	300
*speB*-R		ATTTGAGCAGTTGCAGTAGC	49.7		
*speC*-F	*speC*	CATCTATGGAGGAATTACGC	49.7	55	246
*speC*-R		TGTGCCAATTTCGATTCTGC	49.7		
*speH*-F	*speH*	AAGCAAATTCTTATAATACAACC	46.4	52	630
*speH*-R		TTAGCTGATTGACACATCTACA	49.2		
*speJ*-F	*speJ*	GATAGTGAAAATATTAAAGACG	45.5	52	639
*speJ*-R		GCTCCTATCTTATTTAGTCC	47.7		
*speK*-F	*speK*	GTGTGTCTAATGCCACCGTCT	54.4	56	564
*speK*-R		GGAACATATATGCTCCTAGAT	48.5		

## Data Availability

The data presented in this study are not publicly available as a matter of confidentiality. However, these data are available upon request from the corresponding author.

## Supplementary Materials

The following are available online at https://www.mdpi.com/article/10.3390/biomedicines10040799/s1: Figure S1. The examples of pictures showing results of real-time PCR (amplification curves) for detection of the following genes: *speA*, *speB*, *speC*, *speH*, *speJ*, *speK*. Figure S2. The examples of melting curves and melting peaks pictures showing specificity of the real-time PCR product for the confirmation of the following genes detection: *speA*, *speB*, *speC*, *speH*, *speJ*, and *speK*. Figure S3. An example of the gel picture showing electrophoretic resolution of bacterial DNA and the corresponding PFGE patterns for the selected group of strains. Figure S4. An example of the gel picture showing electrophoretic resolution of bacterial DNA amplification with conventional PCR, the study performed additionally to confirm the sizes of the amplification product for a particular genes and specificity of the applied real-time PCR methodology (*speA*, *speH*, and *speJ* genes). Figure S5. An example of the gel picture showing electrophoretic resolution of bacterial DNA amplification with conventional PCR, the study performed additionally to confirm the sizes of the amplification product for a particular genes and specificity of the applied real-time PCR methodology (*speB*, *speC*, and *speK* genes). Table S1. The detailed origin of the examined *S. pyogenes* strains (units). Table S2. The detailed origin of the examined *S. pyogenes* strains (clinical specimen).
