# Supplementary material for: Assessment of the Relationship between Clinical Manifestation and Pathogenic Potential of Streptococcus pyogenes Strains-Distribution of Genes and Genotypes of Toxins
Source: Biomedicines. 2022 Mar 29;10(4):799. doi: 10.3390/biomedicines10040799 (PMC9029580; doi:10.3390/biomedicines10040799)
Supplement: Supplementary file 1 [file biomedicines-10-00799-s001.zip › biomedicines-1605829-supplementary.pdf]

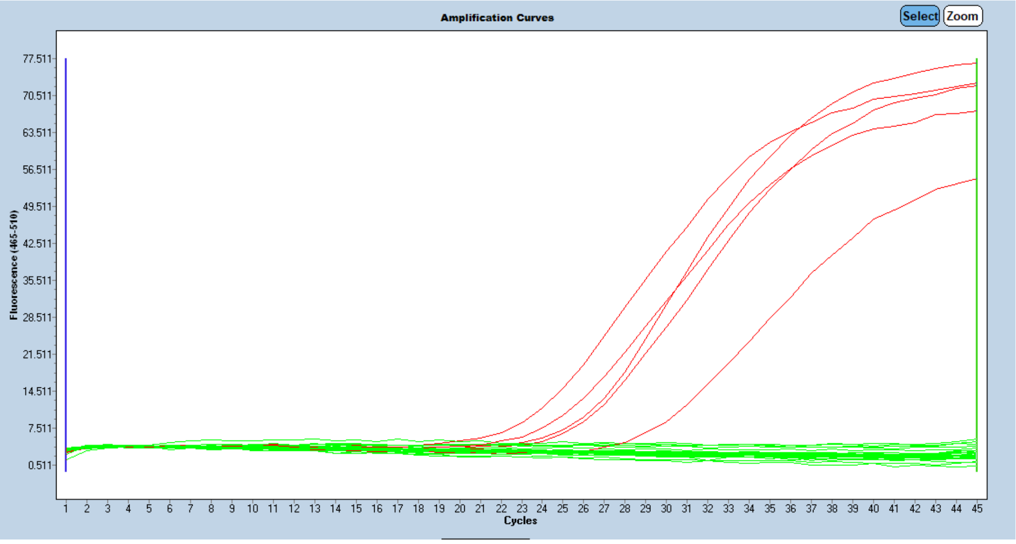
Amplification Curves
Select
Zoom
Fluorescence (465-510)
77.511
70.511
63.511
56.511
49.511
42.511
35.511
28.511
21.511
14.511
7.511
0.511
1 2 3 4 5 6 7 8 9 10 11 12 13 14 15 16 17 18 19 20 21 22 23 24 25 26 27 28 29 30 31 32 33 34 35 36 37 38 39 40 41 42 43 44 45
Cycles

(a)

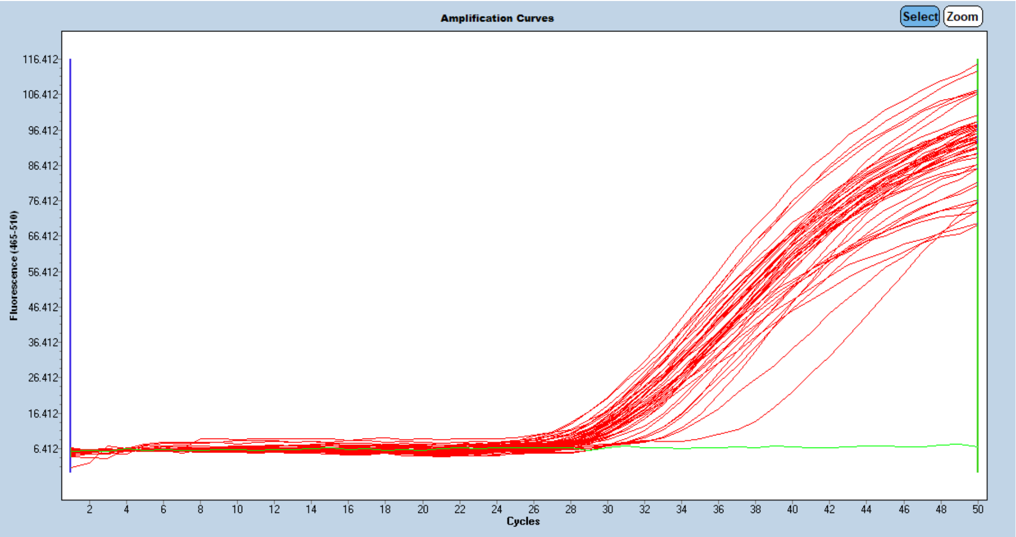
Amplification Curves
Select
Zoom
Fluorescence (465-510)
116.412
106.412
96.412
86.412
76.412
66.412
56.412
46.412
36.412
26.412
16.412
6.412
2 4 6 8 10 12 14 16 18 20 22 24 26 28 30 32 34 36 38 40 42 44 46 48 50
Cycles

(b)

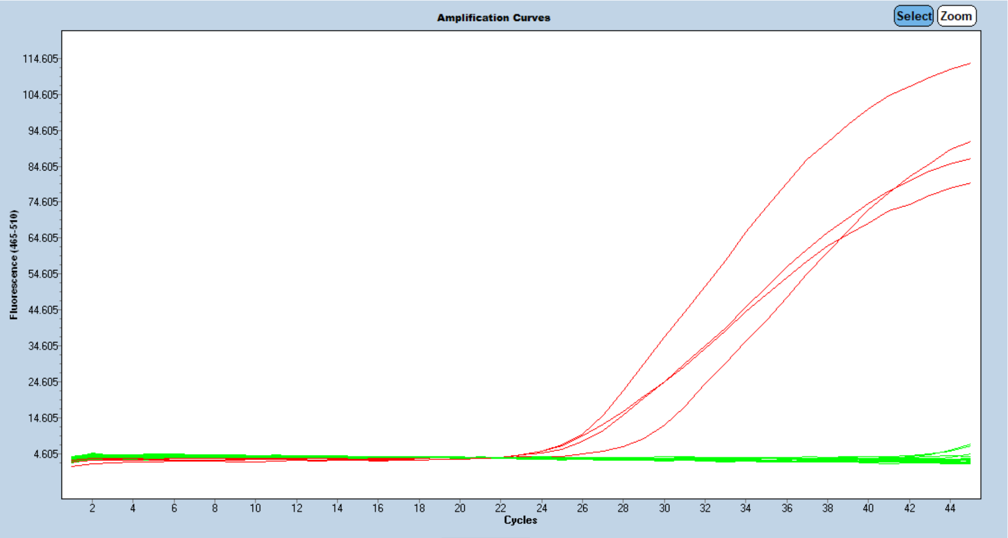
Amplification Curves
Select
Zoom
Fluorescence (465-510)
114.605
104.605
94.605
84.605
74.605
64.605
54.605
44.605
34.605
24.605
14.605
4.605
2 4 6 8 10 12 14 16 18 20 22 24 26 28 30 32 34 36 38 40 42 44
Cycles

(c)

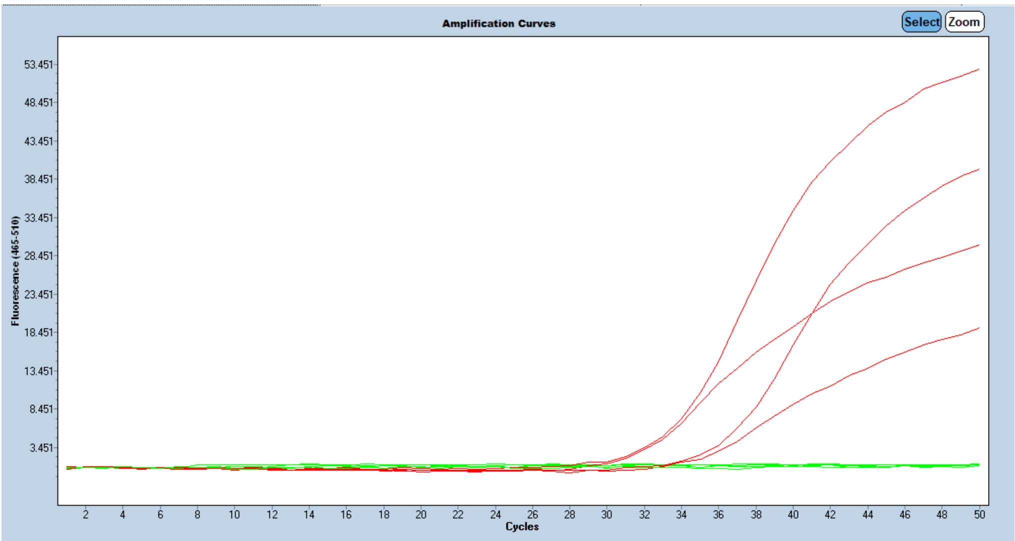

(d)

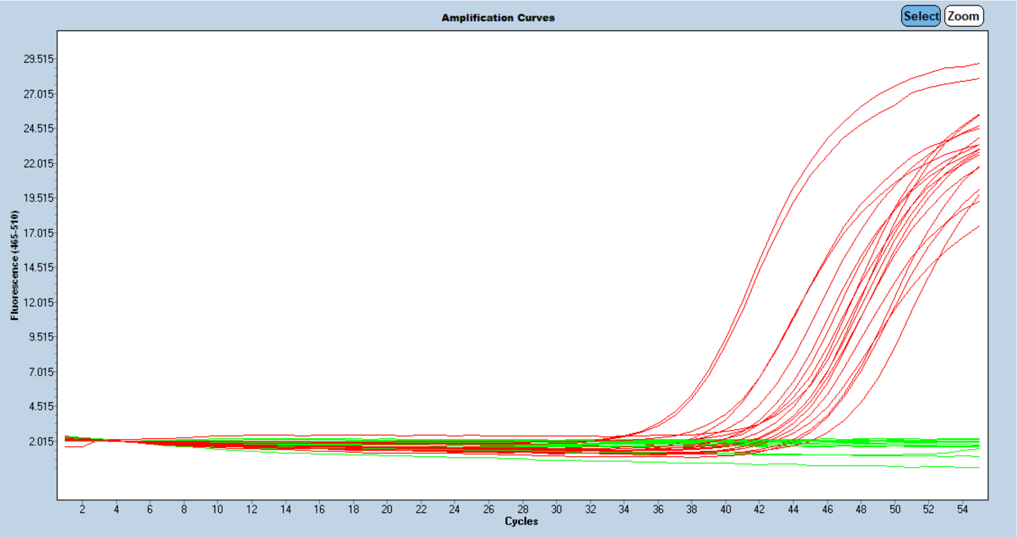

(e)

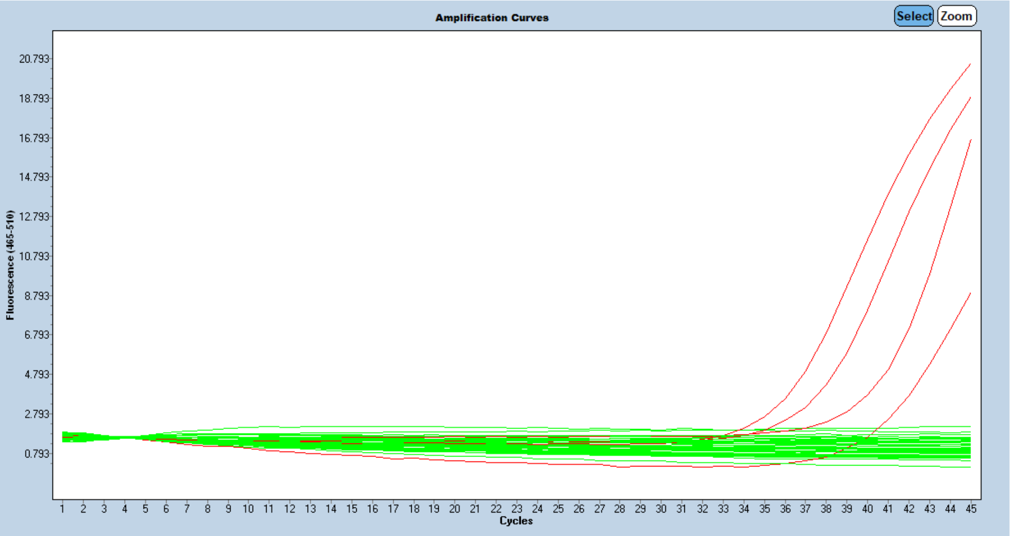

(f)

Figure S1. The examples of pictures showing results of real-time PCR (amplification curves) for detection of *speA* gene (a), *speB* gene (b), *speC* gene (c), *speH* gene (d), *speJ* gene (e) and *speK* gene (f); (red curves – positive results and positive control; green curves – negative results and negative control) .

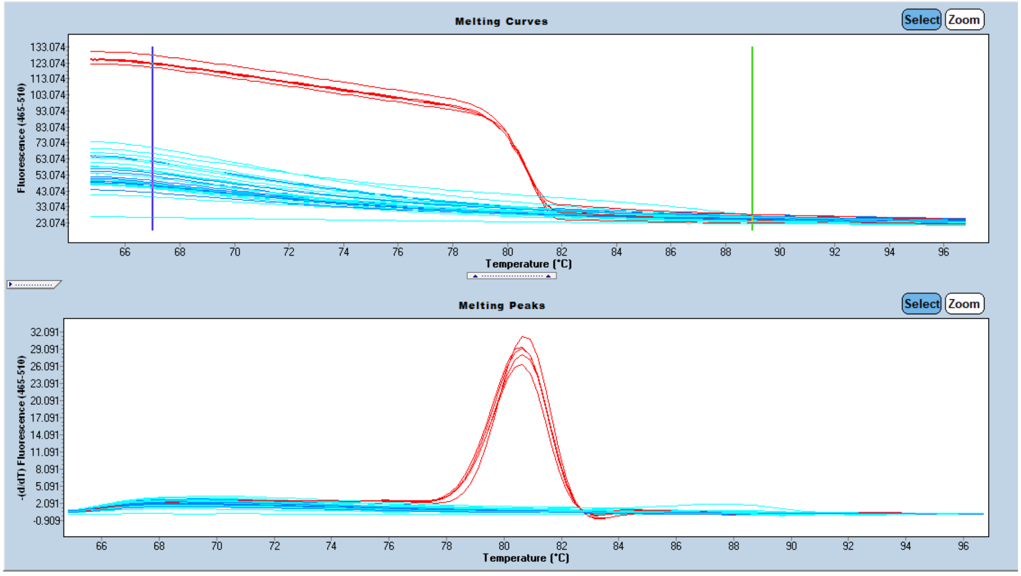
Melting Curves
Select
Zoom
Fluorescence (465-510)
133.074
123.074
113.074
103.074
93.074
83.074
73.074
63.074
53.074
43.074
33.074
23.074
66 68 70 72 74 76 78 80 82 84 86 88 90 92 94 96
Temperature (°C)
Melting Peaks
Select
Zoom
-(d/dT) Fluorescence (465-510)
32.091
29.091
26.091
23.091
20.091
17.091
14.091
11.091
8.091
5.091
2.091
-0.909
66 68 70 72 74 76 78 80 82 84 86 88 90 92 94 96
Temperature (°C)

(a)

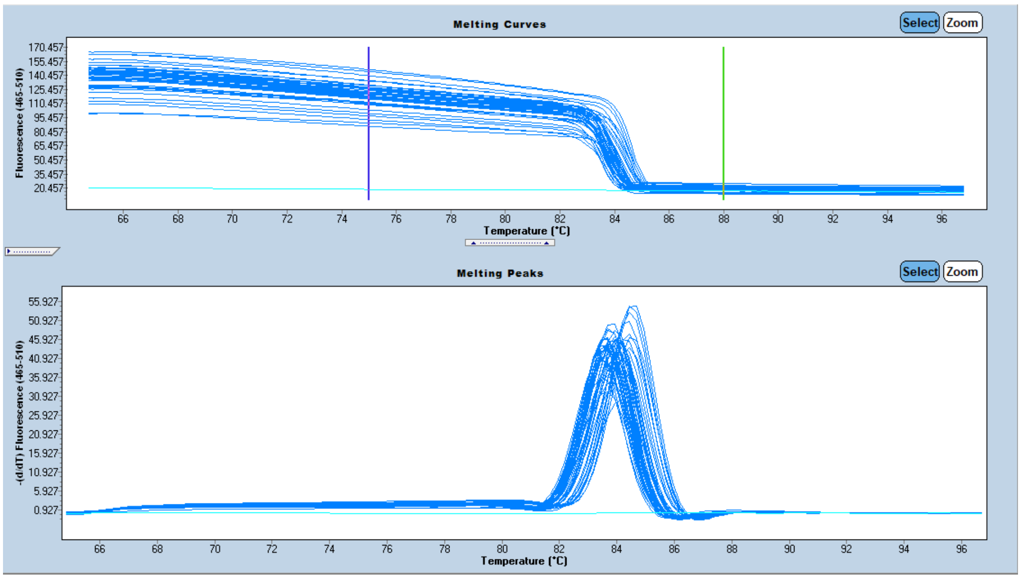
Melting Curves
Select
Zoom
Fluorescence (465-510)
170.457
155.457
140.457
125.457
110.457
95.457
80.457
65.457
50.457
35.457
20.457
66 68 70 72 74 76 78 80 82 84 86 88 90 92 94 96
Temperature (°C)
Melting Peaks
Select
Zoom
-(d/dT) Fluorescence (465-510)
55.927
50.927
45.927
40.927
35.927
30.927
25.927
20.927
15.927
10.927
5.927
0.927
66 68 70 72 74 76 78 80 82 84 86 88 90 92 94 96
Temperature (°C)

(b)

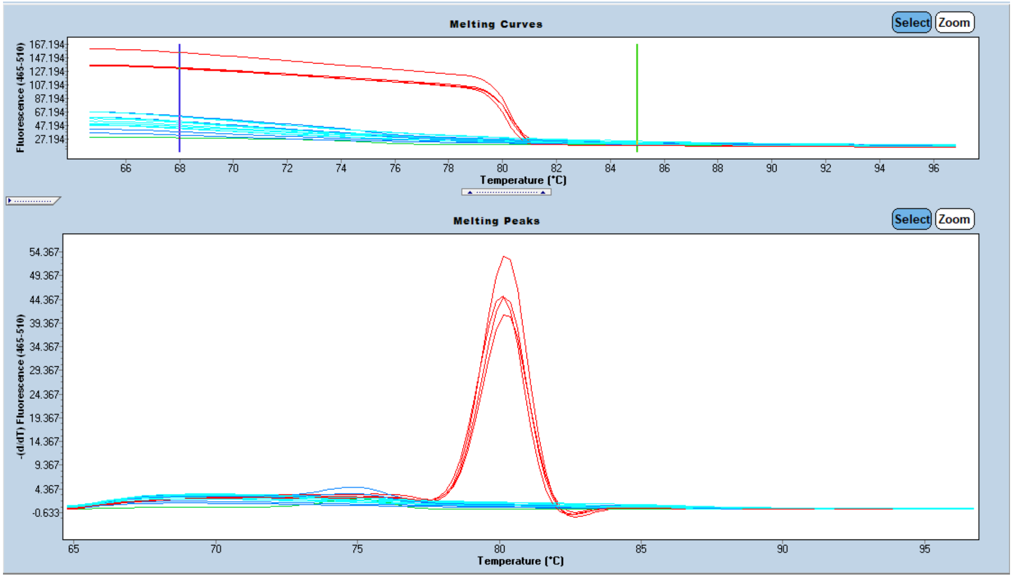
Melting Curves
Select
Zoom
Fluorescence (465-510)
167.194
147.194
127.194
107.194
87.194
67.194
47.194
27.194
66 68 70 72 74 76 78 80 82 84 86 88 90 92 94 96
Temperature (°C)
Melting Peaks
Select
Zoom
-(d/dT) Fluorescence (465-510)
54.367
49.367
44.367
39.367
34.367
29.367
24.367
19.367
14.367
9.367
4.367
-0.633
65 70 75 80 85 90 95
Temperature (°C)

(c)

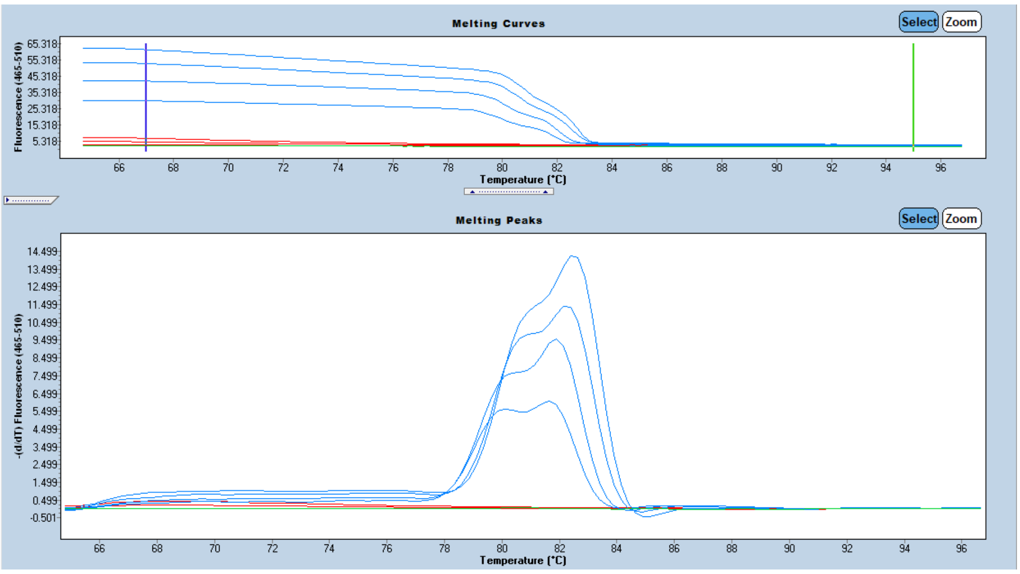

(d)

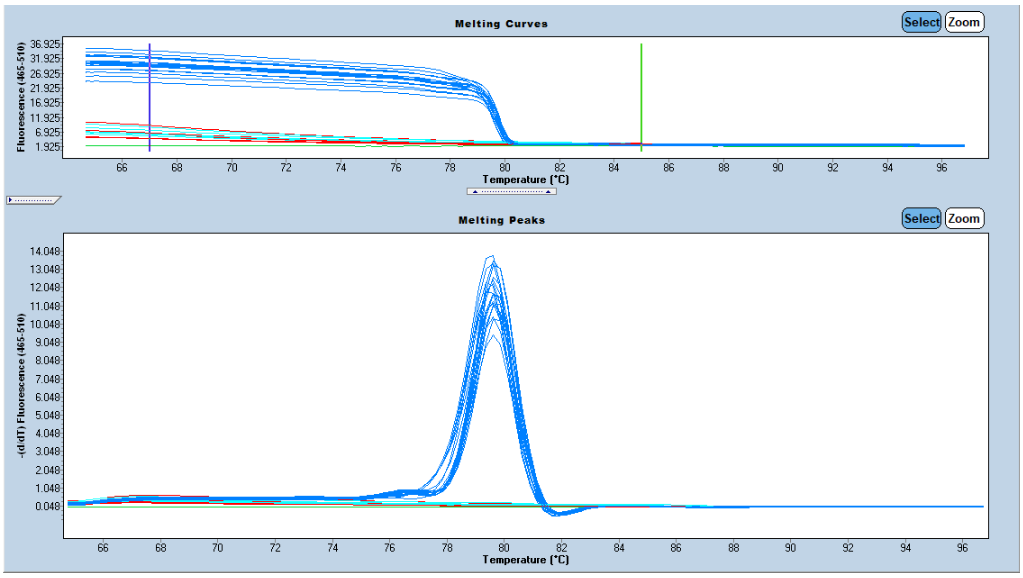

(e)

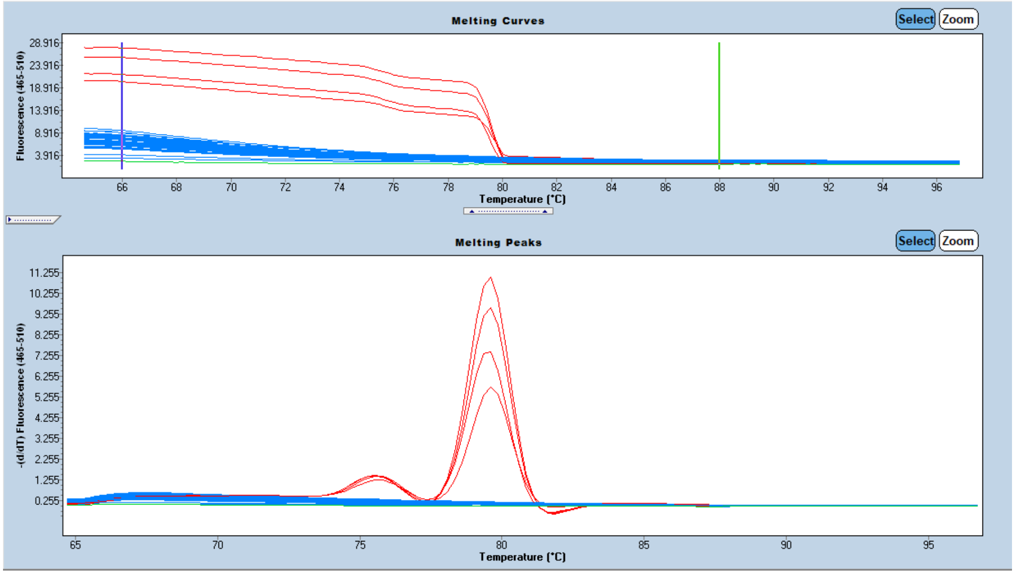

(f)

Figure S2. The examples of melting curves and melting peaks pictures showing specificity of the real-time PCR product for the confirmation of the following genes detection: of *speA* (a), *speB* (b), *speC* (c), *speH* (d), *speJ* (e) and *speK* (f); (red lines - a, c, f, blue lines - b, d, e) .

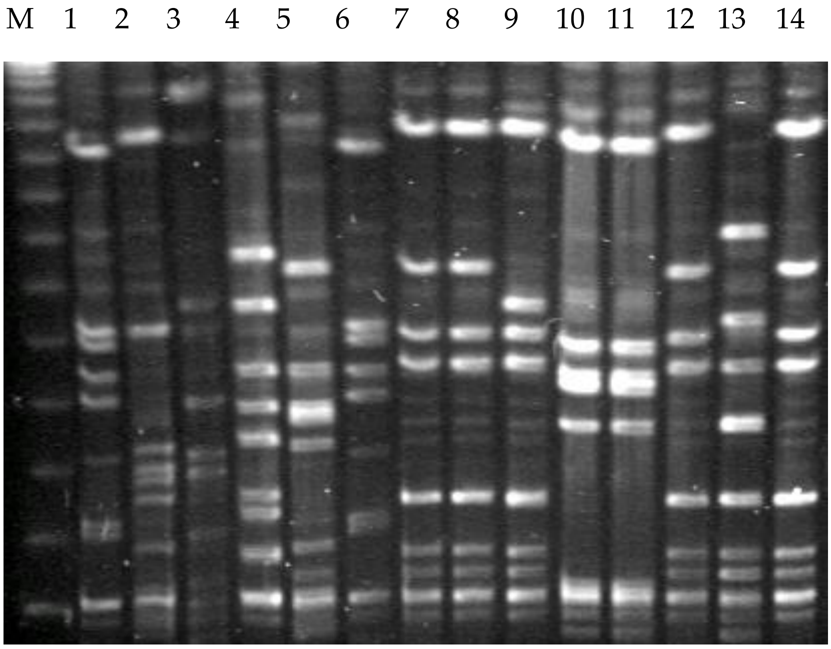

Figure S3. An example of the gel picture showing electrophoretic resolution of bacterial DNA and the corresponding PFGE patterns for the selected group of strains; 1-14 numbers of the examined strains, M – DNA size marker .

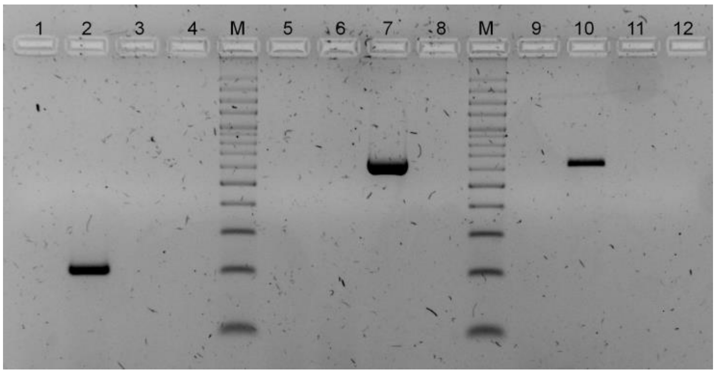

Figure S4. An example of the gel picture showing electrophoretic resolution of bacterial DNA amplification with conventional PCR, the study performed additionally to confirm the sizes of the amplification product for a particular gene and specificity of the applied real-time PCR methodology; 1-4 *speA* gene – 200 bp, 5-8 *speH* gene – 630 bp, 9-12 *speJ* gene – 639 bp, M – DNA size marker (100-3.000 bp) .

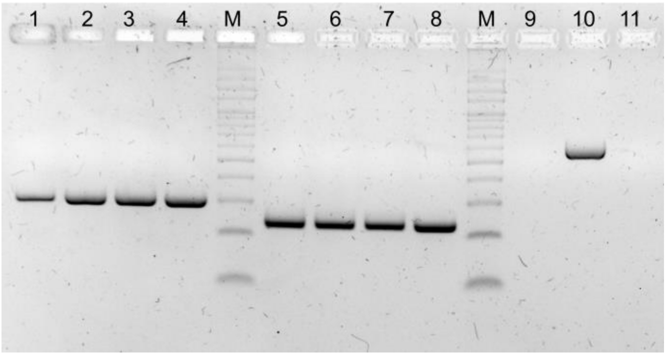

Figure S5. An example of the gel picture showing electrophoretic resolution of bacterial DNA amplification with conventional PCR, the study performed additionally to confirm the sizes of the amplification product for a particular gene and specificity of the applied real-time PCR methodology; 1-4 *speB* gene – 300 bp, 5-8 *speC* gene – 246 bp, 9-11 *speK* gene – 564 bp, M – DNA size marker (100-3.000 bp).

Table S1. The detailed origin (units) of the examined *S. pyogenes* strains (initial group of 168 isolates).

Name of the unit and the number of isolates per particular unit		Number of strains (n = 168)	%
Surgery Departments	Department of Liver and General Surgery – 33	54	32.0
	Department of Vascular Surgery and Angiology – 10		
	Department of General and Oncological Paediatric Surgery – 4		
	Department of Neurosurgery, Neurotraumatology and Paediatric Neurosurgery – 3		
	Department of Orthopaedics and Traumatology – 3		
Outpatient and external		47	28.0
Clinics	Trauma and Orthopedic – 7	18	10.7
	Paediatric Surgery – 4		
	Surgery – 3		
	Paediatric Hematology and Oncology – 1		
	Paediatric Gastroenterology – 1		
	Nutrition – 1		
	Dermatology and Venerology – 1		
Dermatology Department		10	6.0
Otolaryngology	Clinic – 5	9	5.4
	Department – 4		
Department of Cardiology		8	4.8
Department of Nephrology, Hypertension and Internal Medicine		6	3.6
The rest	Department of Anaesthesiology and Intensive Care – 3	16	9.5
	Department of Endocrinology and Diabetology – 3		
	Department of Emergency Medicine – 2		
	Department of Forensic Medicine – 2		
	Department of Paediatrics, Haematology and Oncology – 1		
	Department of Geriatrics – 1		
	Department of Neurology – 2		
	Department of Paediatrics, Allergology and Gastroenterology – 1		
	Department of Ophthalmology – 1		

Table S2. The detailed origin (clinical specimen) of the examined *S. pyogenes* strains (initial group of 168 isolates) .

**Specimen**	***n* = 168**	**%**
Wound swab	65	38.7
Throat swab	33	19.6
Pus samples	20	11.9
Sore swab	13	7.7
Blood samples	13	7.7
Nose swab	7	4.2
Skin swab	3	1.8
The rest: ear swab - 2, tissue - 2, skin lesion swab – 2, stool sample – 1, rectal swab - 1, broncho-alveolar lavage – 1, vaginal swab – 1, eye swab - 1, urethral swab - 1, pleural fluid – 1, catheter outlet swab – 1	14	8.3
